# High frequency activation data used to validate localization of cortical electrodes during surgery for deep brain stimulation

**DOI:** 10.1016/j.dib.2015.11.057

**Published:** 2015-12-11

**Authors:** Efstathios D. Kondylis, Michael J. Randazzo, Ahmad Alhourani, Thomas A. Wozny, Witold J. Lipski, Donald J. Crammond, R. Mark Richardson

**Affiliations:** a Brain Modulation Laboratory, Department of Neurological Surgery, University of Pittsburgh School of Medicine, United States; b Department of Neurobiology, University of Pittsburgh School of Medicine, United States; c Center for the Neural Basis of Cognition, University of Pittsburgh, United States

## Abstract

Movement related synchronization of high frequency activity (HFA, 76–100 Hz) is a somatotopic process with spectral power changes occurring during movement in the sensorimotor cortex (Miller et al., 2007) [1]. These features allowed movement-related changes in HFA to be used to functionally validate the estimations of subdural electrode locations, which may be placed temporarily for research in deep brain stimulation surgery, using the novel tool described in Randazzo et al. (2015) [2]. We recorded electrocorticography (ECoG) signals and localized electrodes in the region of the sensorimotor cortex during an externally cued hand grip task in 8 subjects. Movement related HFA was determined for each trial by comparing HFA spectral power during movement epochs to pre-movement baseline epochs. Significant movement related HFA was found to be focal in time and space, occurring only during movement and only in a subset of electrodes localized to the pre- and post-central gyri near the hand knob. To further demonstrate the use of movement related HFA to aid electrode localization, we provide a sample of the electrode localization tool, with data loaded to allow readers to map movement related HFA onto the cortical surface of a sample patient.

## Specifications Table

**Table:** 

Subject area	*Electrocorticography*
More specific subject area	*Stereotactic methods*
Type of data	*Excel Table, Matlab Interactive Software Package*
How data was acquired	*Electrocorticography, Fluoroscopy, MRI, CT*
Data format	*.xlsx of analyzed data and pivot table, Matlab software package*
Experimental factors	*Human subjects underwent ECoG recordings while squeezing grip force transducers in an externally cued, bilateral hand grip task*
Experimental features	*Activation weight in the high frequency band* (*70*–*100 Hz*) *were calculated from power spectra of ECoG signals during 500 ms epochs before, during and after contralateral hand*
Data source location	*UPMC Presbyterian Hospital, Dept. of Neurological Surgery, Brain Modulation Laboratory*
Data accessibility	*Data are available in accompanying.zip folder, and the localizer sample software package will be maintained on our lab website* (http://www.neurosurgery.pitt.edu/research/brain-modulation-lab)

## Value of the data

• These data validate previous findings that movement related HFA is highly focal, occurring somatotopically in sensorimotor cortex during movement.
• The data and sample localization software package demonstrate movement related HFA is useful for functionally validating the accuracy of electrode localization.

## 1 Data

The data include an excel spreadsheet containing the HFA activation weights for all trials from all patients, bipolar electrode pairs and task epochs. Electrode pairs are reported as the locations (frontal, precentral, postcentral, and parietal) of each electrode, and the task epochs are reported from −4 to 4, with the “0” epoch centered at the onset of movement. This spreadsheet also includes a pivot table for categorical indexing of data. Using this pivot table, readers can explore how high frequency activity is modulated by time relative the onset of movement, and electrode location relative to the central sulcus.

Also included is a sample of the localization tool used to determine the locations of the electrodes reported in the table. The Matlab software package in this sample includes the full functionality described in Randazzo et al. [2], and has a CT skull reconstruction, MRI brain reconstruction and lateral fluoroscopic image pre-loaded. The skull, brain and fluoro image are pre-aligned, but readers can adjust alignment and manually re-register the electrode locations. Additionally, the software package comes with the HFA data for this sample patient pre-loaded and is equipped to map this data onto the cortical surface based upon the electrode localization. This way, readers can explore how the data maps onto the cortex based upon their electrode re-registration. A compiled version of this software package is also included for readers who do not have Matlab installed.

## 2 Experimental design, materials and methods

Study subjects were recruited from a population of patients with Parkinson׳s disease or essential tremor scheduled to undergo implantation of a deep brain stimulator. All subjects were recommended for surgery by their respective multidisciplinary review board based on standard clinical indications and inclusion/exclusion criteria. Informed consent was obtained prior to surgery under a protocol approved by the Institutional Review Board (IRB Protocol #PRO13110420).

The task was conducted intraoperatively with subjects in a semi-seated position in front of a 24′′ computer monitor positioned for comfortable, unobstructed viewing. Subjects held a grip force transducer in each hand and performed an instructed-delay, visually cued, monetary incentive hand grip task. Each trial began with illumination of a yellow light on a traffic light and a hand on one side of the screen indicating with which hand the subject would subsequently squeeze. This instructional cue remained illuminated for 1000–2000 ms, after which the yellow light was replaced with a green light (Go Cue) or red light (No Go cue). Following Go Cues, subjects successfully completed the trial if they responded with the correct hand only in <2 s and maintained at least 10% of their previously measured maximum voluntary grip force for at least 100 ms. Each trial was followed by a feedback message and variable inter-trial delay.

Local field potential (LFP) signals were recorded from subdural cortical strip electrodes temporarily implanted through the burr hole used for DBS implantation [3]. Either 1×6 (*n*=5), 1×8 (*n*=2) or 2×14 (*n*=1) contact platinum iridium cortical strip electrodes (Ad-Tech, Medical Instrument Corporation, Racine, WI) were used. A referential montage was used with a needle reference electrode placed at the left mastoid and a needle ground electrode placed on the shoulder, and signals were subsequently re-referenced offline in a bipolar montage. The 3-dimensional locations of each cortical strip electrode on the cortical surface were estimated using a novel tool described in Randazzo et al. [2].

The task paradigm was implemented using Psychophysics Toolbox on a portable computer. Force signals from the grip force transducers and triggers marking the presentation times of visual cues were digitally recorded simultaneously with ECoG signals. Grip force onsets were calculated offline by smoothing force signals (15 ms running average) and using a 50 N/s threshold to detect changes in the rate of force generation. These grip force onsets were used to segment ECoG data, and the triggers were used isolate successful, contralateral squeeze Go trials. Segmented trial data were further separated into 500 ms long epochs shifted by 250 ms from −1000 ms to 1000 ms relative to grip onset. This resulted in a total of 9 trial epochs with 50% overlap, including 4 pre-movement epochs, 4 post-movement epochs and 1 mixed epoch. A pre-movement baseline epoch for each trial was segmented by capturing the 500 ms preceding the Instruction cue. The activation weight in the high frequency band (70–100 Hz) was calculated from the PSD of the segmented data for each electrode and each trial using a previously described method [1].
