# Supplementary figures and images for: High frequency activation data used to validate localization of cortical electrodes during surgery for deep brain stimulation
Source: Data Brief. 2015 Dec 11;6:204–7. doi: 10.1016/j.dib.2015.11.057 (PMC4707179; doi:10.1016/j.dib.2015.11.057)

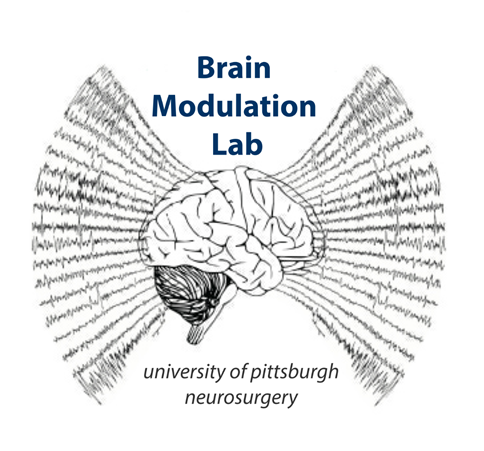

Supplement: Supplementary file 1 — Supplementary material [file mmc1.zip › DBS Localizer Neuroimage/Software Package/Compiled Software Package/Localizer/splash.png]

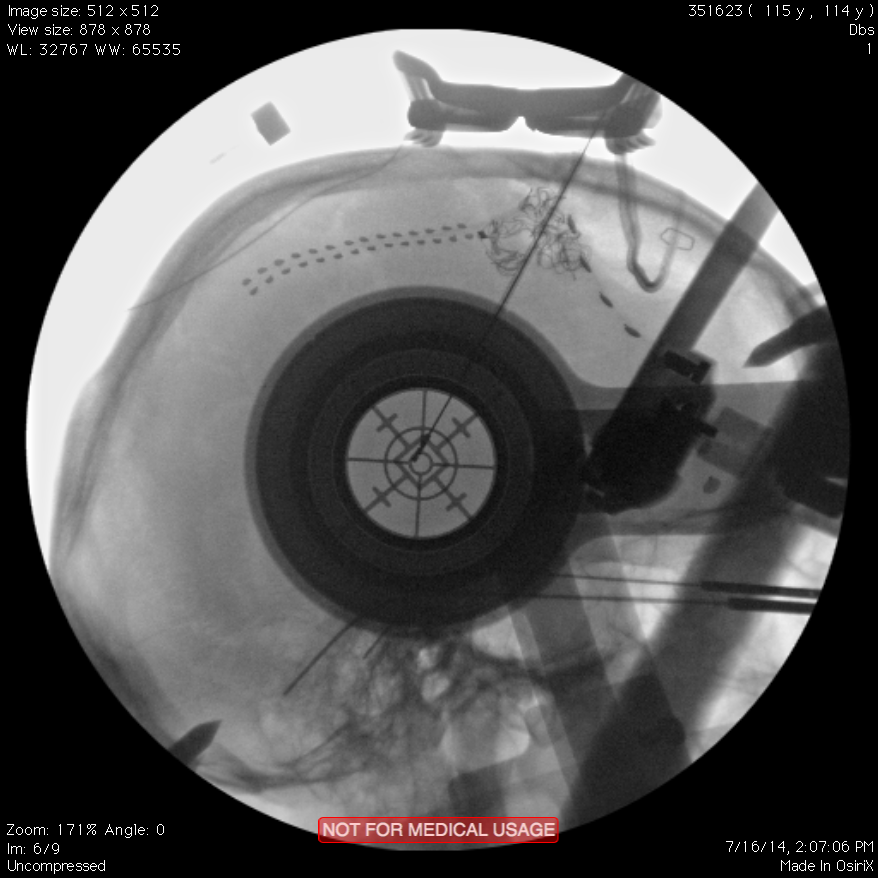

Supplement: Supplementary file 1 — Supplementary material [file mmc1.zip › DBS Localizer Neuroimage/Software Package/Matlab Software Package/LoadFiles/fluoro.tif]
